# Click-Functionalization of Silanized Carbon Nanotubes: From Inorganic Heterostructures to Biosensing Nanohybrids

**DOI:** 10.3390/molecules28052161

**Published:** 2023-02-25

**Authors:** Gririraj Manoharan, Petra Bösel, Jannis Thien, Michael Holtmannspötter, Laura Meingast, Mercedes Schmidt, Henning Eickmeier, Markus Haase, Janina Maultzsch, Martin Steinhart, Joachim Wollschläger, Matteo Palma, Carola Meyer

**Affiliations:** 1 Department of Physics, University of Osnabrück, 49076 Osnabrück, Germany; 2 Department of Chemistry, Queen Mary University of London, London E1 4NS, UK; 3 Center for Cellular Nanoanalytics, University of Osnabrück, 49076 Osnabrück, Germany; 4 Institute of Condensed Matter Physics, Friedrich-Alexander-Universität Erlangen-Nürnberg (FAU), 91058 Erlangen, Germany; 5 Department of Chemistry, University of Osnabrück, 49076 Osnabrück, Germany

**Keywords:** carbon nanotubes, functionalization, silanization, click chemistry, SPAAC, nanohybrids, nano biosensor

## Abstract

Here we present an approach to functionalize silanized single-walled carbon nanotubes (SWNTs) through copper-free click chemistry for the assembly of inorganic and biological nanohybrids. The nanotube functionalization route involves silanization and strain-promoted azide–alkyne cycloaddition reactions (SPACC). This was characterized by X-ray photoelectron spectroscopy, scanning electron microscopy, transmission electron microscopy, Raman spectroscopy and Fourier transform infra-red spectroscopy. Silane–azide-functionalized SWNTs were immobilized from solution onto patterned substrates through dielectrophoresis (DEP). We demonstrate the general applicability of our strategy for the functionalization of SWNTs with metal nanoparticles (gold nanoparticles), fluorescent dyes (Alexa Fluor 647) and biomolecules (aptamers). In this regard, dopamine-binding aptamers were conjugated to the functionalized SWNTs to perform real-time detection of dopamine at different concentrations. Additionally, the chemical route is shown to selectively functionalize individual nanotubes grown on the surface of silicon substrates, contributing towards future nano electronic device applications.

## 1. Introduction

Carbon nanotubes (CNTs) possess unique structural, chemical and physical properties [1] that make them suitable candidates for different applications, including in opto- and bio-electronics [2,3,4,5,6,7,8], advanced composite materials [9,10,11] and biological/biomedical applications [12,13,14,15]. Pristine CNTs have the tendency to aggregate in solution, which limits materials design and solution-processable applications [16,17,18,19]. However, chemical modification by functionalizing CNT surfaces widens the scope to utilize them in different fields such as catalysis, energy storage and nano electronic devices, especially nanoscale biosensors [20,21,22,23]. 

In this regard, functionalized carbon nanohybrids provide access to both the large surface area necessary for gas/liquid–solid interactions and an extended interface, where charge and energy transfer processes can create synergistic effects. Therefore, there is great interest in optimizing CNT functionalization strategies for electrical, optical, mechanical and biological applications [24,25]. To alter the nanotubes into functional nanohybrids, there are three primary functionalization strategies: covalent, non-covalent functionalization and endohedral filling. Non-covalent functionalization involves (bio)macromolecules or wrapping with polymers, whereas covalent functionalization via a *grafting to* or *grafting from* route is the technique used to alter the surface characteristics of the nanotube; both approaches aim to achieve not only better dispersion but also further interactions with functional molecules. Endohedral functionalization is also significant, as the inside of carbon nanotubes is a unique, ultra-pure space, where new reactions may occur [17,26,27].

However, the advanced processing of functional carbon nanomaterials is still restricted because of the complexity of incorporating a wide range of highly engineered molecules onto the surface of CNTs. This challenge can be due to the conflict between reaction environments such as harsh conditions, longer reaction time etc., and functionality of the molecules. Therefore, there is a necessity for a chemical route to functionalize the carbon nanotubes not only in a simple and efficient way but also with high selectivity and relatively mild conditions [28].

Click chemistry can allow us to tailor a wide range of molecules not only in a simple way but also with high efficiency. There are different varieties of click reactions which include Diels–Alder [29], photo click [30] and tetrazine cycloaddition [31] reactions, including the Huisgen azide–alkyne 1,3 cycloaddition [32]. Among these reactions, Cu(I) catalyzed azide–alkyne 3 + 2 cycloaddition (CuAAC) is one of the most studied and effective [32,33,34,35,36,37] and has been incorporated with carbon nanotubes to tailor their properties such as hydrophobicity, conductivity and bio compatibility [36,38,39]. Moreover, covalently functionalized CNTs have been used for sensing small molecules with thin-film transistor devices, as well as for immunoassays with high stability and sensitivity [40,41,42]. There has been a growing interest to develop novel click reactions involving living systems or biomolecules without metal catalysis because of their cytotoxicity [43,44,45,46]. One of the effective yet bio compatible reactions is the copper-free [3 + 2] Huisgen cycloaddition, also known as strain-promoted alkyne–azide cycloaddition (SPAAC) [47]. Though the SPAAC reaction is slower when compared to the CuAAC reaction, it does not compromise high selectivity and efficiency especially with the feasibility in bio-orthogonal labeling and imaging applications [48].

Carbon nanotubes (CNTs) can be functionalized to form a variety of heterostructures, such as CNT–nanoparticle hybrids and bio-CNT nanohybrids [49,50,51,52,53]. These carbon nanohybrids have potential uses in sensing, nanomedicine and catalysis due to their high surface-to-volume ratio and ability to provide sensitivity and electrical connection [24,54,55]. In particular, the integration of carbon nanotubes with gold nanoparticles to produce nanohybrids has gained significant interest in various fields, including biosensors for detecting DNA, proteins and glucose [56,57]. Bio-CNT hybrids with aptamers show promise because of their non-destructive, label-free, real-time electrical detection with high specificity and affinity towards a wide range of entities [19,58,59,60]. Functionalized CNTs can be used in sensing platforms including miniaturized hardware through two main methods: surface growth CNT-lithography and immobilization of solution-processable CNT functionalization. It is desirable to have a synthetic route that can be implemented both in solution and on substrate.

Among the different CNT architectures, single-walled carbon nanotubes (SWNTs) are seamless cylinders of graphene with exceptional thermal conductivity and electronic transport along with high surface area and nanoscale architecture. Depending on their helical angle, they can be semiconducting or metallic. This unique combination of properties possessed by SWNTs makes them a valuable material for use in a wide range of electronic applications, including transistors, interconnects, nano biosensors and microelectronic devices [61,62,63].

Here, we present a strategy of general applicability for the covalent functionalization of SWNTs with various nano moieties of interest, such as metal nanoparticles (gold nanoparticles), fluorescent dyes (Alexa Fluor 647) and DNA aptamers (dopamine-binding aptamers). This was achieved via CNT silanization and strain-promoted azide–alkyne cycloaddition (SPAAC), i.e., copper-free “click chemistry”. We further demonstrate that this covalent approach can also be used to functionalize CVD (chemical vapor deposition)-grown nanotubes directly on substrates. The reaction is specific and selective, thus enabling click functionalization after CNT integration in electronic devices.

## 2. Results and Discussion

**Figure 1:**
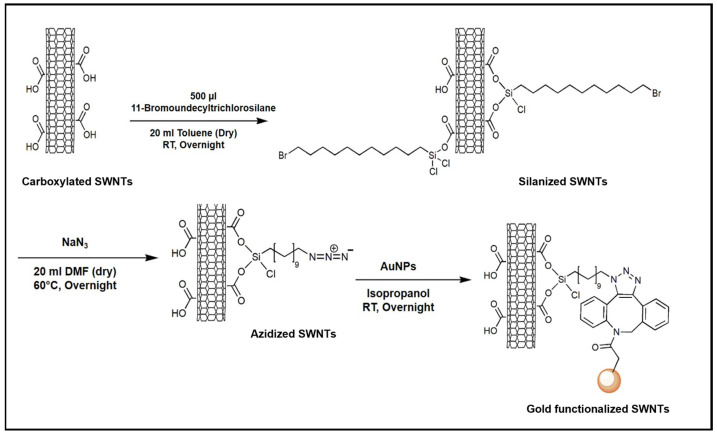
Schematic of all steps of the SWNT functionalization with AuNPs-DBCO. The carboxylated SWNTs are silanized. DBCO is grafted to the azidized silane using SPAAC.

We first functionalized the as-purchased carboxylated single-walled carbon nanotubes with silane in toluene (see Materials and Methods). To initiate the azidization process, sodium azide was mixed with silanized nanotubes in DMF (N, N dimethylformamide) as a one-pot reaction which opens the gate to perform the SPAAC (strain-promoted alkyne–azide cycloaddition) reaction. Lastly, DBCO (dibenzocycloctyne)-terminated gold nanoparticles in IPA (isopropanol) were added to the azidized nanotubes. Figure 1 depicts the general scheme of all functionalization steps.

**Figure 2:**
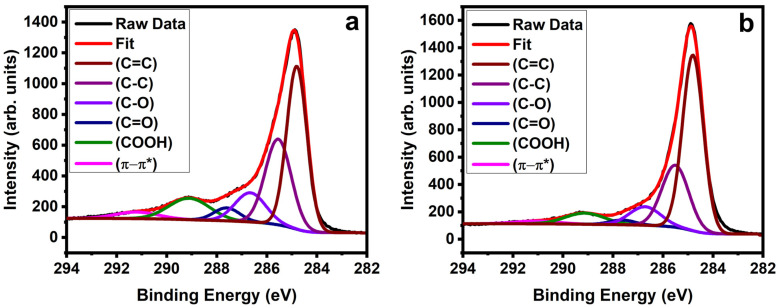
XPS of the carbon C 1s peak of carboxylated SWNTs (**a**) before and (**b**) after silanization.

Characterizations of the functionalized nanotubes were carried out after every reaction with different characterization techniques to better understand the synthetic route. Figure 2 shows the high-resolution C 1s XPS spectra for the carboxylated SWNTs before (Figure 2a) and after (Figure 2b) silanization. The spectra for the carboxylated SWNTs deconvolute into six components: the peak at 284.8 eV (C=C) is attributed to graphitic structure, the peak at 285.5 eV is attributed to sp^3^ hybridized carbon atoms (C–C), and the remaining peaks are 286.6 eV (ether/alcohol C–O), 287.6 eV (carbonyl groups C=O) and 289.1 eV (carboxyl groups COOH). The last peak at 291.2 eV is assigned to the *π* − π* transition peak [64,65,66,67]. All binding energies are calibrated to the sp^2^ hybridized carbon at 284.8 eV.

**Figure 3:**
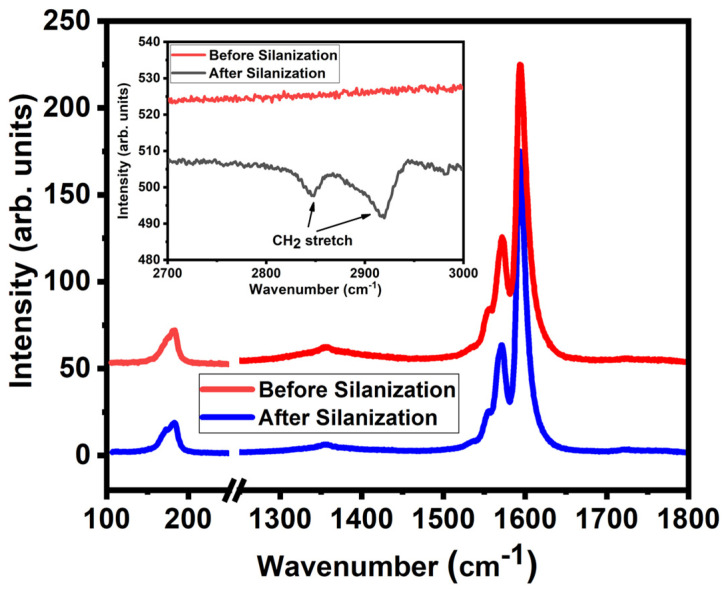
Raman shifts of radial breathing mode, D mode and G mode before and after silanization. Excitation wavelength: 473 nm. Spectra are normalized with respect to G-mode intensity. Inset: FT–IR before and after silanization.

The relative intensity of the COOH peak is large compared to previous reports, while the sp^3^ carbon content is slightly increased [64,66]. We attribute this to the high contribution of carboxylic groups leading to an increase in sp^3^ carbon content as reported by Lee et al. [65]. The COOH peak shows a drastic decrease from 11% to 6.6% after silanization. We attribute this to the successful silanization reaction whereby the silane molecules attached to the functional groups present in the nanotubes. A similar reduction of the COOH peak is reported by Ma et al. [68] after reduction and silanization of MWNTs. Raman investigations were performed to understand before and after silanization reactions, as they tend to give detailed surface information. Figure 3 shows the Raman spectra of both carboxylated SWNTs and silanized SWNTs. It shows the radial breathing modes (RBMs) at around 180 cm^−1^, the D mode at around 1350 cm^−1^ and the G modes at around 1600 cm^−1^ [69]. There is no significant change in the defect-induced D mode after silanization. The silane attachment to the carboxylated SWNTs thus does not lead to additional defects. Previous studies reported a vanishing radial breathing mode (RBM) after functionalization for small-diameter nanotubes [70,71]. In contrast, we do observe RBMs in the Raman spectra before and after silanization. We attribute this to the larger diameter of our SWNTs. Yet, it indicates that the RBM of the carboxylated SWNT, as the D mode, is not strongly affected by the attachment of the silane to the COOH groups. The FT–IR spectra in the inset of Figure 3 show clear absorption peaks at 2918 and 2848 cm^−1^ for the silanized SWNTs, which are caused by the symmetric and the asymmetric stretching mode of methylene groups from the silane [66,67,68]. These modes are absent in the carboxylated SWNTs before the silanization and thus are not caused by aliphatic defects of the tubes as in [72].

**Figure 4:**
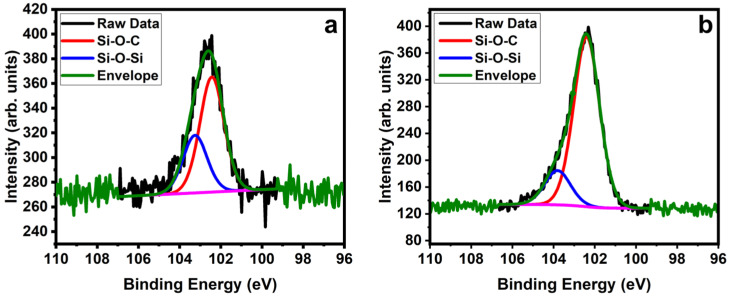
XPS of the Si 2p peak of carboxylated SWNTs after silanization using (**a**) toluene and (**b**) DMF as solvent.

To understand the density of silane functionalization better, we analyzed the XPS spectra of Si 2p spectra. Figure 4 shows high-resolution XPS spectra of the Si 2p signal after silanization. They deconvolute into two peaks that correspond to two chemical states of Si: the peak at 102.4 eV is caused by (Si–O–C) links, while the peak at 103.2 eV shows (Si–O–Si) connections [72]. The former represents the bond of the silane to the SWNT; the latter indicates a cross-linking between silane molecules. Cross-linking is likely between trichlorosilane molecules and is similar as in trimethoxy silanes bound to MWNTs [73]. The ratio between the peaks is thus an indicator for the binding efficiency of the silane to the SWNTs. We find a ratio of about 1:2 for (Si–O–Si)/(Si–O–C) when toluene is used as solvent (Figure 4a). This ratio changes significantly when DMF is used as solvent (Figure 4b), where the ratio is 1:5, indicating a significant increase in silanization efficiency.

**Figure 5:**
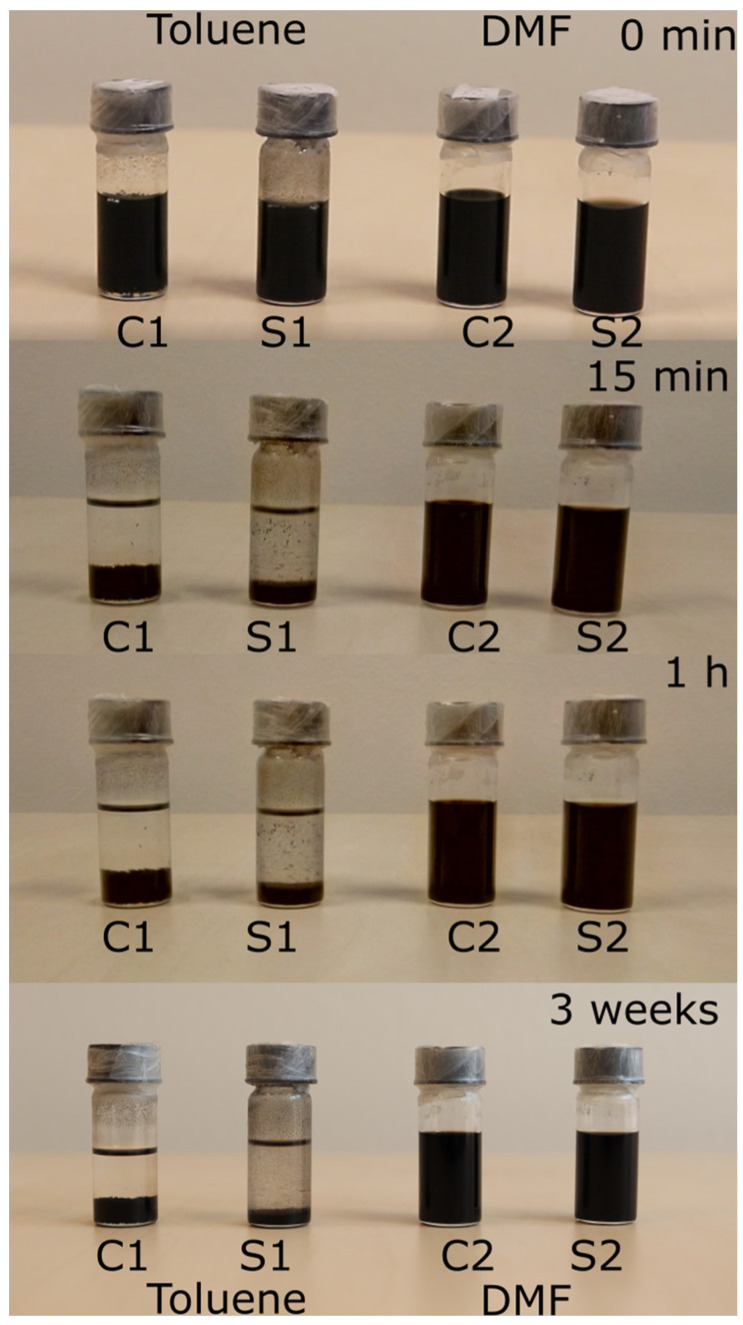
Photographs of carboxylated and silanized SWNTs dispersed in both toluene and DMF.

The different binding efficiency is reflected in the dispersibility of the SWNTs presented in Figure 5, showing four vials containing equal volumes and masses of carboxylated SWNTs and silanized SWNTs dispersed by different solvents (toluene: C1, S1; and DMF (extra dry): C2, S2) after ultrasonication for 30 min. The nanotubes dispersed in toluene quickly settle, though the fraction appears to be lower for the silanized CNTs. We observe the formation of bundles in the supernatant, indicating inefficient silanization in toluene. On the other hand, the carboxylated as well as the silanized SWNTs form a very clear, dark black solution in DMF that remains stable even for several weeks. Here, the two samples show no difference. In summary, the results of the different characterization techniques lead to the conclusion that the silane molecules are grafted to the SWNTs via the carboxyl groups.

**Figure 6:**
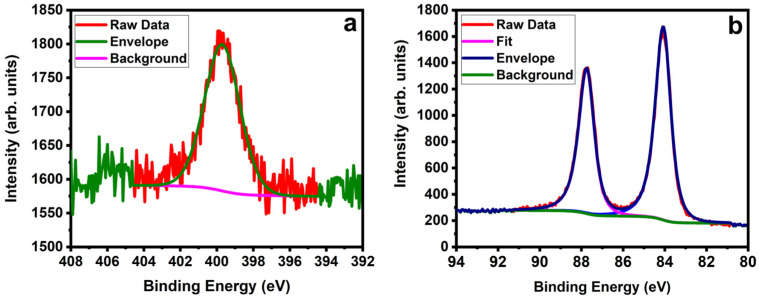
(**a**) High-resolution XPS spectra of N 1s after azidization. (**b**) High-resolution XPS spectra of Au 4f_7*/*2_ and Au 4f_5*/*2_ after AuNPs functionalization of the SWNTs.

We then characterized the following steps of the synthetic route, azidization and click reaction with AuNPs. Figure 6a shows the high-resolution N 1s peak of SWNTs after azidization; it is consigned around 400 eV, which assures nitrogen presence and matches with previous studies [74,75]. The high-resolution C 1s spectra after azidization are shown in the supplementary Figure S1. They show a small shift of the peak formerly attributed to carbonyl groups from 287.6 to 288 eV. We attribute this to the presence of C–N bonds after azidization [76]. The successful click reaction with AuNPs-DBCO is shown by the high-resolution XPS Au 4f spectra in Figure 6b. The Au 4f_7*/*2_ and Au 4f_5*/*2_ peaks are assigned at 84.3 eV and 87.9 eV, respectively [57,77,78].

**Figure 7:**
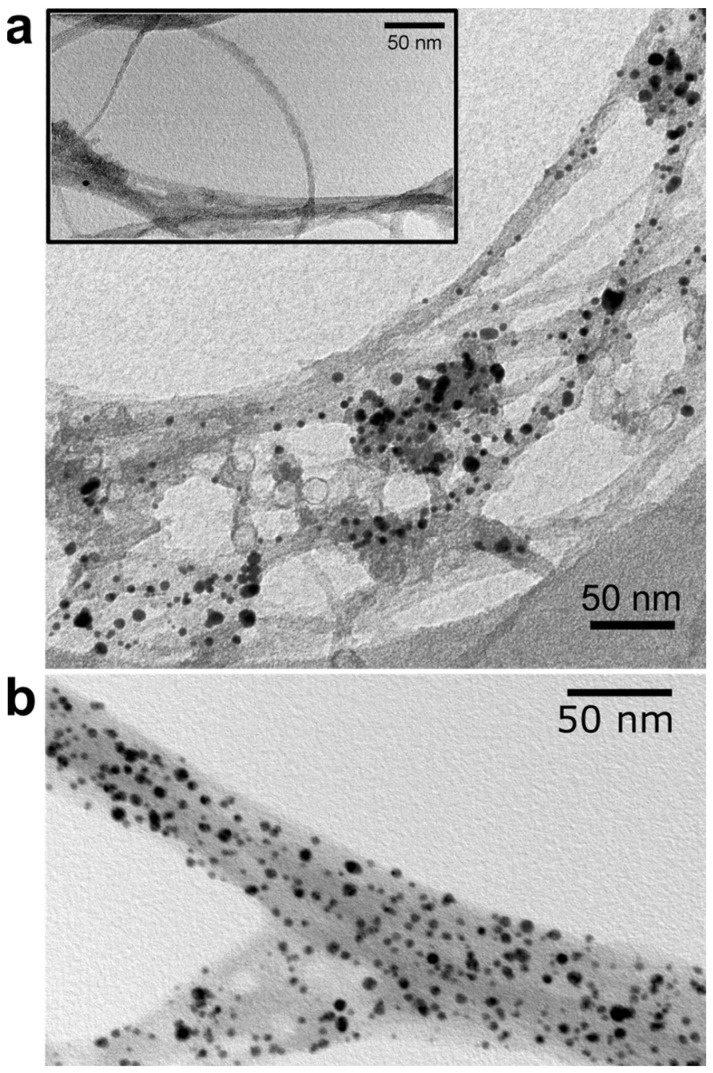
TEM images of Au-functionalized SWNTs with different solvents used during the silanization step: (**a**) toluene (**b**) DMF. The inset shows the negative control sample.

TEM was used for the structural characterization of the gold-functionalized SWNTs. Figure 7 shows images of SWNTs after functionalization using different solvents: gold nanoparticles are clearly identified on the surface of the functionalized SWNTs. The functionalization appears to be rather inhomogeneous when toluene is used as solvent (Figure 7a), comparable to previous studies on MWNTs functionalized with AuNPs [57,78]. We attribute this to the agglomeration of SWNT bundles during the series of reactions. As a result, the components that tend to attach are less likely to react with their corresponding functional groups compared to well-dispersed samples. As a control experiment, the whole synthesis procedure is followed without involving any of the main constituents such as silane or azide. As the final step, the DBCO-functionalized AuNPs are added to the solution followed by the washing procedure.

We did not find any trace of gold nanoparticles on the CNTs in the subsequent TEM characterization, as shown in the inset of the Figure 7a. This implies the specific bonding between azide and DBCO in the actual functionalization. The distribution of gold nanoparticles is much more homogeneous when DMF is used as solvent, as shown in Figure 7b. Mostly, individual AuNPs are attached, though sometimes two of them appear close together. Larger agglomerations appear very rarely. Together with the XPS data of the Si 2p peak, this supports the interpretation that cross-linking between the silanes is the major reason for the strong trend toward agglomeration of the nanoparticles functionalized to the silanized CNTs in toluene. The density of the functionalization is similar to the one achieved by Gobbo et al., [77] who functionalized SWNTs with gold nanoparticles through SPAAC, but with the DBCO attached to the CNT.

Pristine CNTs tend to form bundles when dispersed onto a substrate. Fabrication of electronic devices based on long, individual functionalized SWNTs requires the functionalization to be carried out on tubes directly on a substrate. To demonstrate that our route can be transferred to the substrate, we carried out all functionalization steps of carbon nanotubes on Si/SiO_2_ substrate and optically transparent quartz coverslips.

Carbon nanotubes are grown by CVD on the substrate from dilute catalyst to prevent catalyst residue from covering the surface (Figure S2a; see supplementary information). Surface passivation of the substrate is necessary, since otherwise the SiO_2_ surface of the substrate would be silanized and therefore functionalized along with the CNTs. The passivation layer must sustain oxidation for 30 min in air at T = 450 °C. Therefore, we chose another silane, FDTS (perfluorodecyltrichlorosilane), to passivate the SiO_2_.

**Figure 8:**
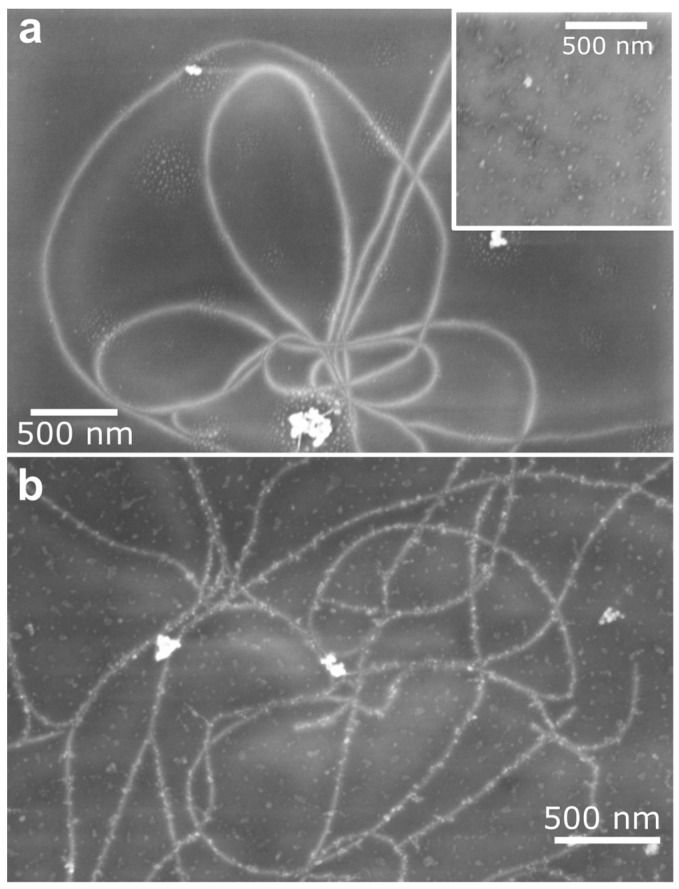
SEM images of CVD-grown nanotubes (**a**) after passivation and oxidation and (**b**) after functionalization with gold nanoparticles. Very bright white aggregated spots indicate catalyst particles. The inset shows the SiO_2_ surface after passivation and oxidation.

The SEM image in Figure 8a shows the sample after the oxidation step. While the CNTs look similar to the image of the pristine tubes, the oxidized FDTS appears as small dots on the surface (inset Figure 8). Figure 8b shows an SEM image taken after the click reaction. Here, the CNTs are clearly covered with gold nanoparticles. The homogeneity and density of the achieved functionalization are similar to the one presented in Figure 7b for samples prepared in solution.

Fluorescent dyes integrated into carbon nanotubes will open a more extensive range of applications, particularly in single-molecule devices based on optics in the field of labeling, nano biosensors and controlled drug delivery. Investigations of photophysical properties of photosensitive molecules attached to SWNTs are interesting, as they make nanohybrid materials for sensing and light-harvesting devices [79,80].

To demonstrate the versatility of the synthetic route, we employed an AF647 (Alexa Fluor 647) fluorescent dye to functionalize the nanotubes in solution. Alexa Fluor 647 is one of the most commonly used clickable fluorescent dyes in advanced microscopic setups because of its brightness, long wavelength and relatively low self-quenching under imaging conditions. Alexa Fluor dyes are spectrally similar to cyanine dyes but have more advantages relatively, such as higher FRET (fluorescence resonance energy transfer) efficiencies, higher quantum efficiency and capability of retaining their fluorescence after functionalization [81].

**Figure 9:**
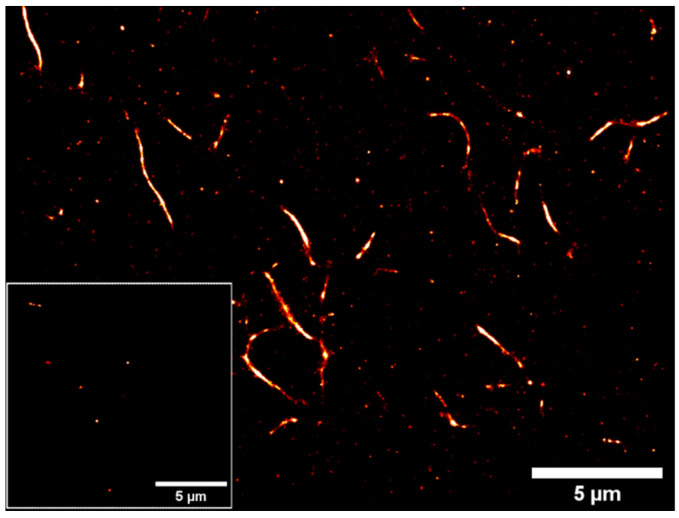
STORM image of AF647-functionalized SWNTs. Inset: Control experiment of nanotubes with AF647 (not functionalized). Imaging conditions: 20K frames recorded. Activation laser wavelength: 405 nm. Excitation laser wavelength: 642 nm.

To localize the fluorophores covalently attached with SWNTs with high accuracy, we employ the STORM (stochastic optical reconstruction imaging) imaging technique. It can identify numerous fluorophores and resolve ultra-structures under 20 nm after the reconstruction of many iterations [76]. Figure 9 shows the STORM images of AF647-functionalized SWNTs drop-cast on the coverslip. The experiment is performed with oxygen-scavenging buffer to control photobleaching of the fluorescent dyes. Frames of 20 K are reconstructed to form a super-resolved image.

We managed to resolve individual nanotube bundles of less than 100 nm diameter and length of around 5 μm, and the grafting efficiency of fluorophores on the CNTs was found to be homogeneous. A high concentration of functionalized SWNTs and free dyes on the surface are imaged, and the residues can be reduced after further dilution. A control experiment was carried out to demonstrate the covalent attachment of fluorescent dyes towards the nanotubes and not just free dyes covering the nanotubes.

Figure 9 inset shows the STORM image of the control experiment with AF647 dyes added to the nanotubes without following the functionalization procedure. As expected, there are slight traces of free dyes on the surface of the coverslip but not from the nanotubes.

**Figure 10:**
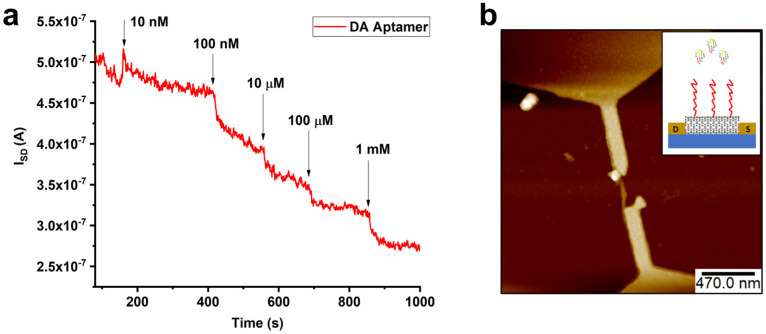
(**a**) Real-time detection of dopamine at various concentrations (10 nM to 1 mM). (**b**) AFM image of the dopamine binding aptamer functionalized CNTFET. Inset: Cartoon of the aptamer-CNTFET.

To show the applicability of the presented strategy for biosensing applications, we functionalized SWNTs with aptamers to fabricate a nanoscale biosensor (see experimental section) and demonstrate the recognition of dopamine at physiological concentrations [82]. Figure 10 shows the real-time detection of dopamine with various concentrations (10 nM to 1 mM) with high sensitivity. A decrease in the electrical response (source (S)–drain (D) current, I_SD_) was observed upon the addition of target analytes. This is in line with the response of similar CNT–aptamer hybrid biosensing devices [19].

A random aptamer (not selective to dopamine) was employed as a control experiment. As we expected, no significant change in the current response of the devices was found upon addition of dopamine, as shown in supplementary Figure S3.

## 3. Materials and Methods

Carboxylated SWNTs were used from Carbon Solutions, Inc., Riverside, CA, USA. A total of 100 mg of nanotubes was sonicated (Bandelin electronic GmbH, Berlin, Germany) with 20 mL of extra-dry toluene (99.85 %, Acros Organics, Darmstadt, Germany) for about 30 min in a 40 mL Falcon tube. After sonication, the suspension was transferred to a new vial to separate the solution from carbon residues. An amount of 500 µL of 11-bromoundecyltrichlorosilane (ABCR GmbH, Karlsruhe, Germany) was added, and the suspension was stirred for 18 h in a pure argon-infused, round-bottom flask, as the silane is sensitive to ambient situations. After silanization, the suspension was centrifuged (Allegra X-15R Centrifuge, Beckman Coulter, Brea, CA, USA) at 4000 RPM for 10 min, and the supernatant was discarded. The suspension was dispersed again with 20 mL of toluene and sonicated for 5 min and centrifuged at 4000 RPM. The washing procedure was carried out three more times with the silanized SWNTs with 20 mL toluene and centrifuged to remove the unreacted silane and amorphous carbon along with the excess solvent present in the suspension. Then, the same washing procedure was continued with 20 mL of N, N dimethylformamide (extra-dry DMF) (Acros Organics, Darmstadt, Germany) twice before the next reaction.

Silanized SWNTs were dispersed in 20 mL of DMF (extra-dry) with a spatula of sodium azide (NaN_3_) (Merck KGaA, Darmstadt, Germany) and stirred in a lab set up oil bath at 60 °C for 18 h in an argon environment. Following the reaction, the SWNTs were sonicated for 5 min before the suspension was vacuum-filtrated (vacuum filtration unit, Sartorius Stedim Biotech, Göttingen, Germany) to separate the azidized SWNTs from excess solvents and residues. The same washing procedure was carried out with 20 mL of distilled water, and the process was repeated thrice. Then the suspension was washed in 20 mL of isopropanol (IPA) (Sigma Aldrich, Taufkirchen, Germany) twice before the final reaction. Gold nanoparticles attached with dibenzocycloctyne (AuNPs-DBCO) (Nanopartz Inc., Loveland, CO, USA) (dry, 1 mg) were dissolved in 1 mL of distilled water, forming a colloid. Azidized SWNTs and AuNPs-DBCO solution were dispersed in 10 mL of IPA, and the suspension was stirred for 18 h at room temperature.

After the click reaction, the suspension was centrifuged at 4000 RPM for 10 min. The supernatant was again discarded before sonicating the remaining suspension with 20 mL of IPA for the further washing process. The same procedure was repeated five times to eradicate the unreacted elements along with excess solvent from the suspension. Finally, the suspension was vacuum-dried to remove the solvent present in the precipitate after the washing procedure.

The route to functionalize the CVD-grown carbon nanotubes on Si/SiO_2_ substrate was almost the same as that of the bulk material, with minor changes. Carbon nanotubes were grown at a growth temperature T = 900 °C for 10 min with methane as the carbon feedstock on a catalyst drop-cast Si/SiO_2_ substrate via the lab CVD set up [83]. The set up comprises a tubular furnace (Heraeus, Hanau, Germany) connected with the controlled gas systems (Hagemann GmbH, Ochtrup, Germany). After growth, the substrate was silanized with thermally stable FDTS (ABCR GmbH, Karlsruhe, Germany) through vapor deposition at 80 °C for 2 h. FDTS acts as a passivation layer for the substrate to ensure selective functionalization of the nanotubes. The silanized substrate with CNTs was then oxidized at 450 °C for 30 min to create carboxylic groups on CNTs with high density, which serve as the binding sites for further functionalization. The density of the functional groups can be controlled by the oxidation time [84]. It has been shown that a low density does not significantly affect the transport properties [85].

After oxidization, the sample functionalization followed the scheme of Figure 1, with conditions adapted for the functionalization on the substrate. Silanization was achieved through vapor deposition at 80 °C for 2 h knowing that the silane will attach to the carboxylic groups created on the surface of the nanotubes. Sodium azide solution was prepared in N, N dimethylformamide (1 mg in 5 mL) under an argon atmosphere and sonicated for 5 min. The following azidization reaction was carried out at 60 °C for 18 h on the substrate. Finally, the gold nanoparticles (0.5 mg dry) were dispersed with distilled water, and the solution was treated with the substrate for 18 h at room temperature. The substrate was washed after the click reaction with water first and then through sonication for 5 min with acetone (Sigma Aldrich, Taufkirchen, Germany) and IPA, respectively. Likewise, the carbon nanotubes were functionalized with fluorescent dyes (AF647) following the same synthetic route until azidization. AF647-DBCO (Jena Bioscience, Thuringia, Germany) was dispersed in DMSO to perform the click reaction on azidized nanotubes from solution. The STORM imaging technique of SWNTs functionalized with AF647 was performed by using a TIRF 4-Line STORM microscope (Olympus (Evident), Tokyo, Japan).

Lastly, the azidized carbon nanotubes from solution-processed nanotubes were functionalized with dopamine-binding aptamers (5AmMC6/GT CTC TGT GTG CGC CAG AGA ACA CTG GGG CAG ATA TGG GCC AGC ACA GAA TGA GGC CC) (Integrated DNA Technologies, Leuven, Belgium) to fabricate the nano biosensor [86]. Azide-functionalized carbon nanotubes were immobilized by drop-casting 5 µl of CNT solution to prepatterned silicon substrate through dielectrophoresis. The parameters of dielectrophoresis were maintained at V_p-p_ = 2 V for 2 min with *f* = 400 KHz in a parameter analyzer (Keithley 4200SCS Tektronix, Beaverton, OR, USA) for the electrode pads in the substrate. Then the devices were washed with water and blown gently with N_2_ gas. A volume of 100 µl of 100 µM amine-terminated, dopamine-binding aptamers was conjugated with DBCO-sulfo-NHS to synthesize DBCO-terminated aptamers. Then, azidized nanotubes were functionalized with DBCO-aptamers for 3 h on substrate. The same procedure was followed with the random aptamers (5AmMC6/GC ATG TAC AAC AAT ATT TAT TAG TCA TCT TTG AGA CAC AAT CTC CCA CCT CAC TGG AA) (Integrated DNA Technologies, Leuven, Belgium) for the control experiments.

Characterization of the samples was carried out by using X-ray photoelectron spectroscopy (XPS) (ESCA-unit Phi 5000 VersaProbe III, ULVAC-PHI Inc.,Kanagawa, Japan), Raman spectroscopy (Horiba LabRam HR Evolution, Kyoto, Japan), Fourier-transform infrared spectroscopy (FT–IR), transmission electron microscopy (TEM) (JEM 2100 plus, JEOL, Tokyo, Japan), atomic force microscopy (AFM) (Bruker Dimension Icon, Bruker, Karlsruhe, Germany) and scanning electron microscopy (SEM) (Zeiss EVO MA 10, Zeiss, Oberkochen, Germany). XPS measurements were implemented under ultra-high vacuum in an ESCA-unit Phi 5000 VersaProbe III with a base pressure of 10^–9^ mbar. Al K*α* radiation of a monochromatized Al anode (h*ν* = 1486.6 eV) was used as the X-ray source. The sample solutions were prepared in IPA and coated on a copper substrate for the measurements. Raman spectroscopy was carried out with a Horiba LabRam HR Evolution confocal Raman microscope, equipped with a 473 nm laser, 600 lines/mm grating and a 100× long-working-distance objective. For all spectra, the laser power was kept at 0.5 mW. For the TEM analysis, JEM 2100 plus was operated at 200 KV and equipped with LaB6 for imaging the gold-functionalized nanotubes. Functionalized SWNTs were examined through TEM to study the post-functionalization characteristics and to recognize the functionalization density. The sample solutions were drop-cast on a holey copper TEM grid (Plano GmbH, Hessen, Germany) for measurements. Dispersibility of the carboxylated SWNTs and silanized SWNTs were measured using two different solvents: toluene and DMF. SWNTs (5 mg) were mixed in 4 mL solvent solution. The mixture was sonicated for 1 h and stored at room temperature.

The topographic analysis of the SWNTs before and after functionalization with aptamers was imaged with Bruker Dimension Icon atomic force microscopy (AFM) using ScanAsyst air tips. Electrical measurements were performed using a four-probe station (PS-100 Lakeshore, Lake Shore Cryotronics, Inc., Westerville, OH, USA) with a parameter analyzer (Keithley 4200SCS, Tektronix, Beaverton, OR, USA) for the real-time dopamine sensing of the functionalized nanotubes with both DBCO aptamers and random aptamers.

## 4. Conclusions

In summary, we have presented an efficient and mild strategy for the functionalization of SWNTs by using a synthetic route involving both silanization and click reactions (SPAAC). Surface characterization (XPS) after every reaction confirmed successful functionalization. The solvent (DMF or toluene) used during the initial process affects the homogeneity and the density of the functionalization. We employed this strategy to then tether AuNPs (5 nm), organic dyes (AF647) and aptamers to CNTs, demonstrating how our route can be easily employed to graft a variety of nanomaterials to SWNTs on different surfaces. The nanoparticle attachment was resolved via SEM, while functionalization with dyes was demonstrated via STORM microscopy. Additionally, we immobilized dopamine-aptamer-functionalized nanotubes on pre-patterned substrates in nanoscale device configurations and demonstrated the sensitive recognition of dopamine. This work lays the foundation for grafting various functional molecules to carbon nanotubes with nanoscale control in both solution and on substrate, contributing towards nanoelectronic device implementation and biosensing applications.

## Data Availability

Not applicable.

## Supplementary Materials

The following supporting information can be downloaded at: https://www.mdpi.com/article/10.3390/molecules28052161/s1, Figure S1: XPS spectra of the C1s spectra (a) after silanization and azidization and (b) after functionalization with gold nanoparticles; Figure S2: SEM images of CVD-grown nanotubes (a) before functionalization and (b) after functionalization with gold nanoparticles. Very bright white spots indicate catalyst particles. Gold nanoparticles are selectively functionalized on the surface of the nanotubes; Figure S3: Real-time sensing of random-aptamer-nanohybrid-functionalized SWNTs with different concentrations. As expected, no significant change was observed upon the addition of dopamine target analytes; Figure S4: AFM images of the CNT-FET (a) before and (b) after functionalization with dopamine-binding aptamers. Significant changes in the height profile of the CNTs were observed after functionalization with aptamers.
