# Supplementary material for: Click-Functionalization of Silanized Carbon Nanotubes: From Inorganic Heterostructures to Biosensing Nanohybrids
Source: Molecules. 2023 Feb 25;28(5):2161. doi: 10.3390/molecules28052161 (PMC10004328; doi:10.3390/molecules28052161)
Supplement: Supplementary file 1 [file molecules-28-02161-s001.zip › molecules-2190335-supplementary.pdf]

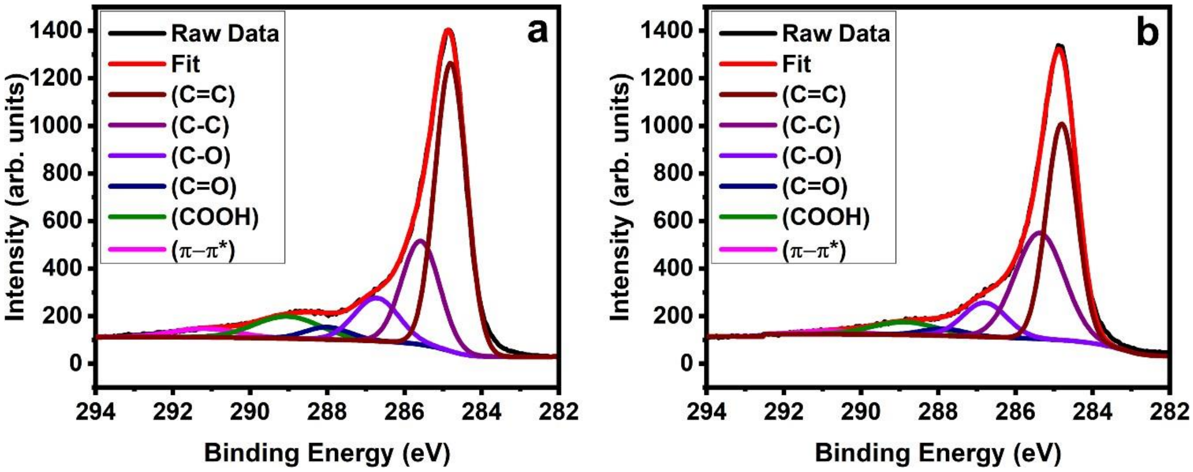

Figure S1. XPS spectra of the C1s spectra (**a**) after silanization and azidization and (**b**)after functionalization with gold nanoparticles.

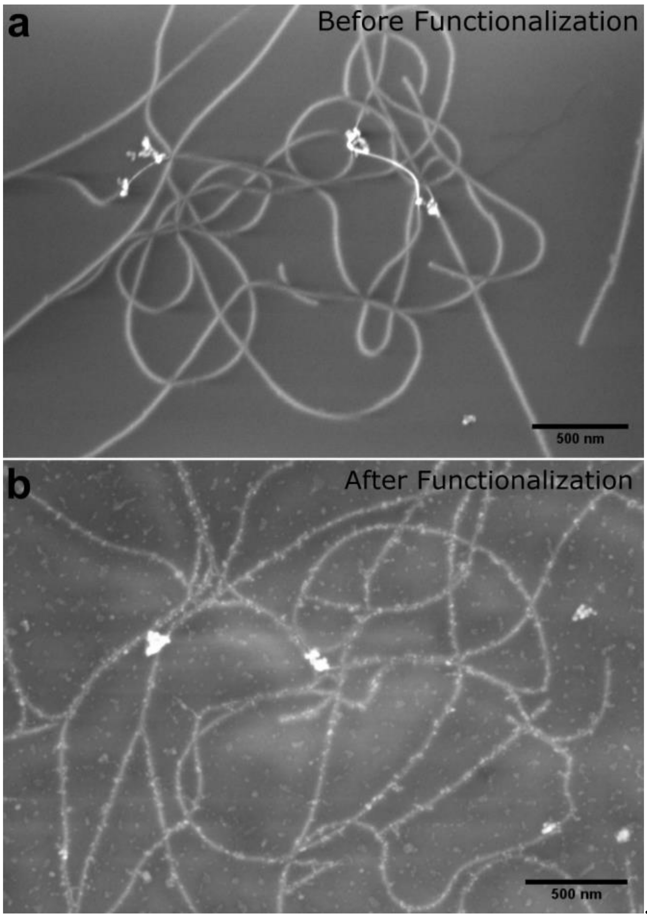

Figure S2. SEM images of CVD grown nanotubes (**a**) before functionalization and (**b**) after functionalization with gold nanoparticles. Very bright white spots indicate catalyst particles. Gold nanoparticles are selectively functionalized on the surface of the nanotubes.

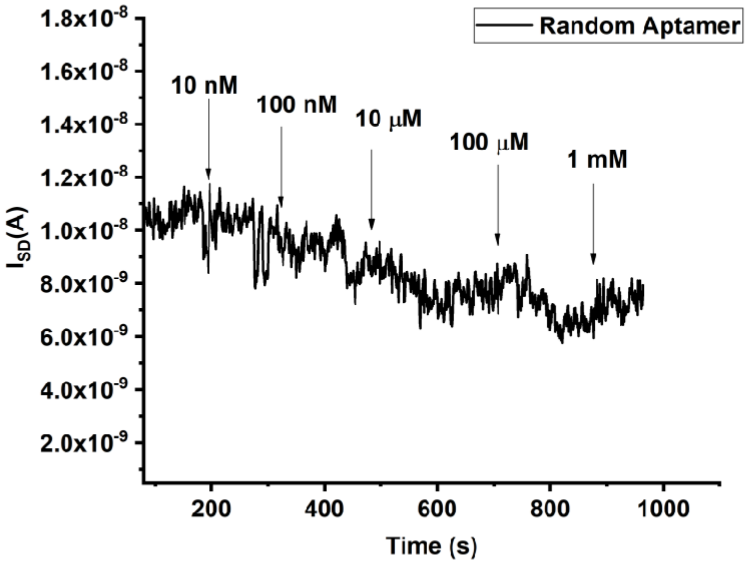

Figure S3. Real-time sensing of random aptamer nanohybrids functionalized SWNTs with different concentrations. As expected, no significant change was observed upon the additionof dopamine target analytes.

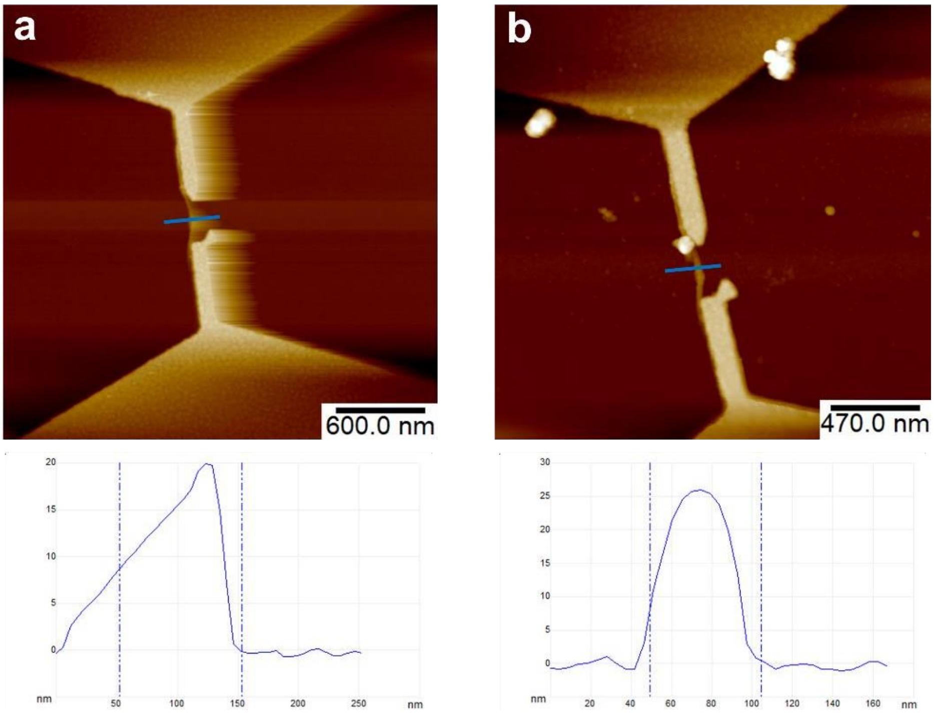

Figure S4. AFM images of the CNT-FET (**a**) before and (**b**) after functionalization with dopamine binding aptamers. Significant change in the height profile of the CNTs were observed after functionalization with aptamers.
